# A Robust, Enzyme-Free Glucose Sensor Based on Lysine-Assisted CuO Nanostructures

**DOI:** 10.3390/s16111878

**Published:** 2016-11-14

**Authors:** Qurrat-ul-Ain Baloach, Aneela Tahira, Arfana Begum Mallah, Muhammad Ishaq Abro, Siraj Uddin, Magnus Willander, Zafar Hussain Ibupoto

**Affiliations:** 1 Kazi Institute of Chemistry, University of Sindh, Jamshoro 76080, Pakistan; qurrat2014@gmail.com (Q.-A.B.); aneelatahira85@gmail.com (A.T.); arfana30@gmail.com (A.B.M.); 2 Department of Metallurgy and Material Science, Mehran University of Engineering and Technology, Jamshoro 76080, Pakistan; ishaque.abro@faculty.muet.edu.pk; 3 National Center of Excellence in Analytical Chemistry, University of Sindh, Jamshoro 76080, Pakistan; drsiraj03@gmail.com; 4 Department of Science and Technology, Campus Norrkoping, Linkoping University, Norrkoping SE-60174, Sweden

**Keywords:** CuO nanostructures, lysine, glucose sensor, electrochemical techniques

## Abstract

The production of a nanomaterial with enhanced and desirable electrocatalytic properties is of prime importance, and the commercialization of devices containing these materials is a challenging task. In this study, unique cupric oxide (CuO) nanostructures were synthesized using lysine as a soft template for the evolution of morphology via a rapid and boiled hydrothermal method. The morphology and structure of the synthesized CuO nanomaterial were characterized using scanning electron microscopy (SEM) and X-ray diffraction (XRD), respectively. The prepared CuO nanostructures showed high potential for use in the electrocatalytic oxidation of glucose in an alkaline medium. The proposed enzyme-free glucose sensor demonstrated a robust response to glucose with a wide linear range and high sensitivity, selectivity, stability, and reproducibility. To explore its practical feasibility, the glucose content of serum samples was successfully determined using the enzyme-free sensor. An analytical recovery method was used to measure the actual glucose from the serum samples, and the results were satisfactory. Moreover, the presented glucose sensor has high chemical stability and can be reused for repetitive measurements. This study introduces an enzyme-free glucose sensor as an alternative tool for clinical glucose quantification.

## 1. Introduction

Exploring the physicochemical features of nano-dimensional materials with controlled morphology, size, and specificity is a challenging and demanding task [1]. Controlling the shape hierarchy of nanomaterials is a new step towards tuning their properties [2]. Among the various transition metal oxides, cupric oxide nanostructures have received great attention from the scientific community for their wide range of applications, including uses in antimicrobials [3,4], chemical and biological sensors [5,6], optoelectronics [7], and photonic and electronic devices [8]. Cupric oxide nanostructures possess desirable properties such as high surface-to-volume ratios, conductivity, and cost effectiveness [3]. The controlled morphology of copper oxide at nanoscale dimensions may significantly affect catalytic, optical, and electrical characteristic and may do so at low cost [9]. CuO nanostructures exhibit a narrow band gap (1.2–1.6 eV) with enhanced catalytic performance and good chemical stability [10]. Different growth techniques have been used to prepare copper oxide nanomaterial with controlled shapes [9,11]. For example, Jiang el al. synthesized different CuO morphologies via hydrothermal methodology with copper acetate in basic pH [12]. Zhang et al. prepared nanoribbon-like mesoporous CuO using a tetraoctylammonium bromide soft template [13]. An urchin CuO nanostructure morphology was achieved by Keyson et al. [14] by exposing alkaline copper carbonate (CuCO_3_·Cu(OH)_2_) in polyethylene glycol to microwave irradiation. These prime and reproducible nanostructures, due to their multi-dimensional properties, such as high surface-to-volume ratios, have the most promising complex functions [9] reported for CuO. However, using self-assembling basic nanodimensional units to control the architecture and shape of CuO nanostructures is a new direction in their synthesis.

Diabetes is a chronic disease that affects millions of people worldwide [9]. Monitoring physiological glucose concentrations is an very important, routine analysis to be performed to avoid health issues such as diabetes [15,16]. Therefore, the rapid and accurate determination of glucose has become a hotspot of research both in clinical diagnosis and biotechnological research fields [17]. Presently, glucose in blood or serum samples is assessed using techniques based on the electrochemical oxidation of glucose using glucose oxidase enzymes, due to their excellent selectivity, accuracy and sensitivity [18,19]. However, the potential capabilities of these devices are limited by the complexity of complex fabrication of electrode fabrication and the poor enzyme stability in harsh environments, the responses of enzyme-based biosensors are affected by moisture in the environment, whether during usage or in storage. Hence, a simple, cost-effective protocol to measure glucose concentrations with high accuracy, both in vivo and vitro, is highly desirable [20]. Cost-effective electrooxidation catalysts have been studied extensively for the development of sensors are highly sensitive, have quick response times, and are acceptably stable and reproducible. Metal or metal oxide-based nanomaterials are often employed in sensor technology due to their excellent surface reactivity and catalytic performance, which is maintained throughout long durations of time, and their rapid electron transfer rate [20]. The disadvantages of enzyme-based biosensors can be overcome easily by using a suitable nanomaterial. Moreover, the desired nanostructures can decrease sensor size, reduce cost, and allow use in the continuous quantification of analytes in urine, blood, or serum samples. Currently, CuO nanostructures are good candidates for these sensing applications due to their improved catalytic effect and resistance to surface poisoning [21]. CuO nanoparticles [22], nanoplatelets [23,24,25,26,27,28,29], nanowires [20], nanoflowers, and nanorods [10] have been used to sense glucose with enhanced sensitivity. Various non-enzymatic glucose sensors have been developed, as well, such as a 3D nanoporous Pt electrode [30], a PtAu/C nanocomposite [31], electrospun Co_3_O_4_ nanofibers [32], Pt nanoparticles that are decorated with carbon nanotubes/TiO_2_ composite [33], and cobalt oxide nanoflowers [34], among others. Many of these nanostructures involve expensive and complex preparations. However, a solution-based approach to synthesize desired nanostructures is the most suitable method to produce a large number of nanostructures with fascinating properties. 

In this work, a simple hydrothermal growth method is used to synthesize CuO nanostructures rapidly, which serve as highly sensitive and selective enzyme-free glucose sensors. The developed enzyme-free glucose sensor exhibited potential electrocatalytic activity towards the oxidation of glucose in NaOH with robust sensing performance. Moreover, the sensors were used to detect glucose levels of fresh serum samples to evaluate their real-time application.

## 2. Materials and Experimental Section

### 2.1. Chemicals

Analytical-grade copper chloride (CuCl_2_·2H_2_O) (99.9%), ammonia solution (NH_3_) (33%), lysine, glucose, uric acid, ascorbic acid, dopamine, and sodium hydroxide were purchased from Sigma-Aldrich (Karachi, Pakistan). Isopropanol (C_3_H_8_O) obtained from Merck, Karachi Pakistan was used to dissolve 1% Nafion (C_7_HF_13_O_5S_·C_2_F_4_) solution (Sigma).

### 2.2. The Synthesis of Lysine-Assisted CuO Nanostructures Using a Rapid Hydrothermal Treatment Method

Lysine-assisted CuO nanostructures were synthesized using low-temperature, aqueous chemical growth methodology. In this preparation method, 100 mL of 0.1 M CuCl_2_·2H_2_O was first homogenized with 1 g lysine. Afterwards, 33% NH_3_, and the growth solution underwent a 2 h hydrothermal treatment in a pre-heated electric oven at 200 °C. The resulting CuO nanomaterial was rinsed with deionized water to remove surface impurities prior to its use in the modification of glassy carbon electrodes. In this study, lysine was used as a soft template for the CuO nanomaterial evolution of morphology.

### 2.3. Characterization and Electrochemical Cell Assembly

Scanning electron microscopy (SEM) (JEOL, Takyo, Japan) and X-ray diffraction (XRD, Bruker D-8, Fischers, New York, NY, USA) were used to study the morphological, structural, and compositional features of the prepared CuO nanostructures. The electrochemical cell assembly used a 760D model electrochemical work station with a calomel reference electrode and a platinum wire counter electrode. A CuO-nanostructure-modified GCE was used as the working electrode. All glucose measurements were performed in 0.1 M NaOH.

### 2.4. Preparation of Modified Glassy Carbon Electrodes (GCEs)

GCE surfaces were modified with CuO nanostructures using the methodology described in our previously published work [24]. Briefly, the GCE was gently polished with 1 μm alumina paste and ultrasonically cleaned in deionized water and ethanol. The drop-casting method was used to modify GCEs using a suspension of CuO nanostructures (0.5 g/mL ethanol). The deposited nanomaterial was coated with 1% Nafion to ensure that the CuO nanostructures firmly adhered. If the prepared CuO nanostructures are directly grown on a conductive substrate using similar growth conditions, this substrate can serve as a working electrode. If the growth of CuO nanostructures on a conductive substrate is successfully established, can be used for the non-enzymatic detection of glucose. 

## 3. Results and Discussion

### 3.1. The Structural and Morphological Characterization of CuO Nanostructures Obtained via Use of Lysine as a Soft Template

**Figure 1:**
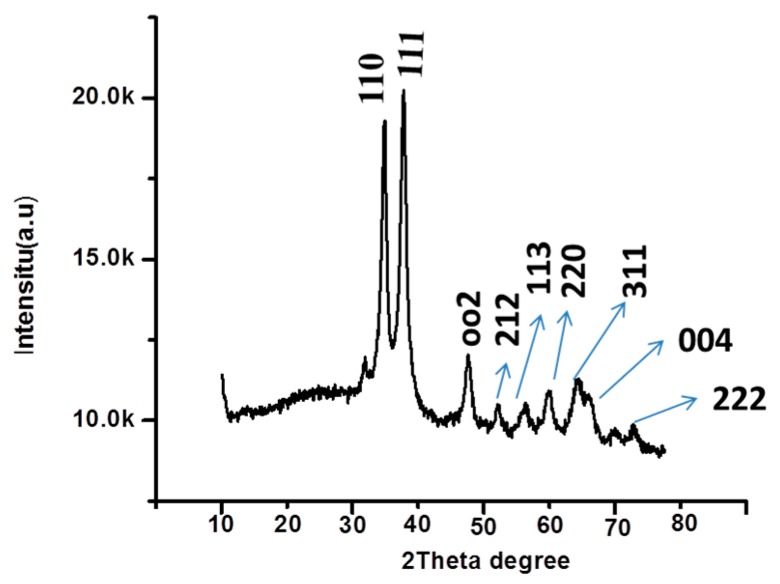
XRD spectrum of CuO nanostructures prepared in the presence of lysine as a soft template.

**Figure 2:**
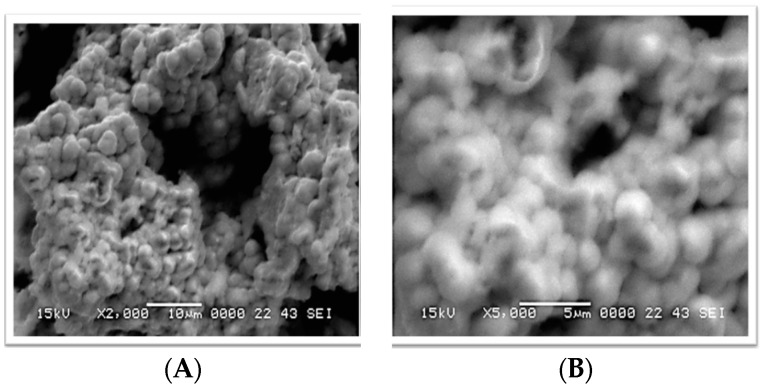
Scanning electron microscope images of CuO nanostructures at (**A**) low and (**B**) high magnification levels.

Figure 1 depicts the measured XRD patterns of the synthesized CuO nanostructures. The diffraction peaks around (110), (111), (002), (212), (113), (220), (311), (004), and (222) were assigned to the pure monoclinic phase of CuO (JCPDS Card No. 45-0937). The prepared CuO nanomaterial was determined to be of high purity and excellent crystalline quality. Figure 2 shows the SEM images of the CuO nanostructures obtained using lysine as a soft template. The exhibited morphology was that of a cotton flower at low and high magnification (Figure 2A,B, respectively). The lysine played a crucial role in tuning the morphology of CuO nanostructures by controlling the dimensions and features.

### 3.2. Electrochemically Sensing Glucose Using Different Electrochemical Modes

**Figure 3:**
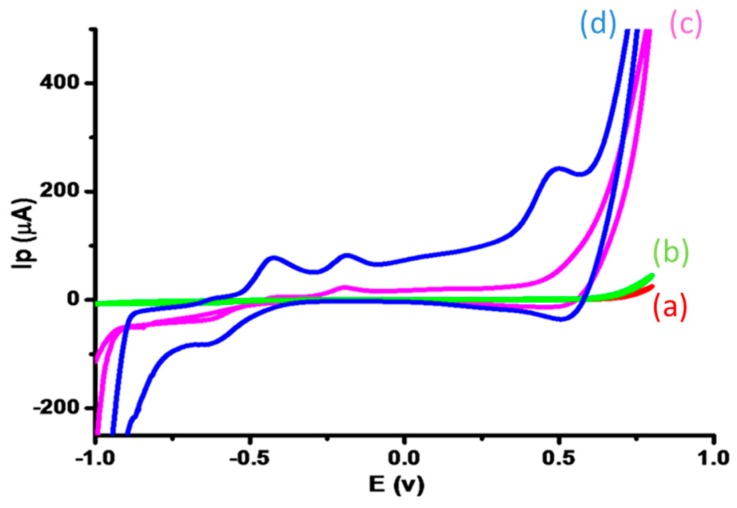
Cyclic voltammograms recorded in 0.1 M NaOH in either (**a**) the absence of glucose or (**b**) the presence of 1 mM glucose with a bare GCE; and CuO-nanostructure-modified GCEs in either (**c**) the absence of glucose or (**d**) the presence of 1 mM glucose. Scan rates are 0.05 mV·s^−1^.

The sensing mechanism of glucose on the surface of CuO nanostructures in alkaline medium was elucidated by modifying the GCE with CuO nanostructures as depicted in Figure 3. Glucose oxidation, over newly prepared CuO nanostructures in NaOH, generated gluconic acid or other intermediate products. The most suitable and possible mechanism for glucose oxidation on at CuO-nanostructure-modified electrodes in a basic medium is shown in the following Equations (1) and (2):

(1) CuO + OH > CuOOH + e^−^

(2) CuOOH + e^−^ + glucose > CuO + OH + gluconic acid

**Figure 4:**
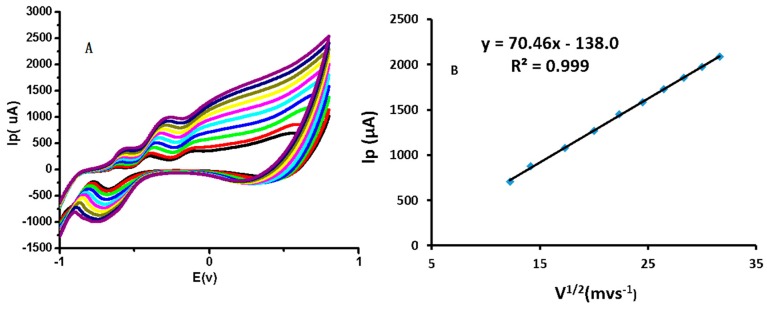
(**A**) Cyclic voltammograms obtained with CuO-nanostructure-modified GCEs in 0.1 M NaOH and 1 mM glucose at various scan rates (150, 200, 300, 400, 500, 600, 700, 800, 900 and 1000 mV·s^−1^; (**B**) The inset shows the plot of Ip versus the square root of the scan rate.

The production of electrons during glucose electrooxidation reactions is apparent in Figure 3, where the CV response of a modified electrode in the presence of 1 mM glucose produces a broader anodic peak around +0.5 V. No redox peak was found in the absence of glucose. The successive increase in current shows well-organized, inherited catalytic features of CuO nanostructures that serve to enhance the sensing performance of the modified electrode, such as a large surface area and rapid electron transfer of as-prepared CuO nanostructures in the current research findings. The electrode response in the presence of 1 mM glucose at various scan rates showed that the reaction at the modified electrode surface was diffusion-controlled (Figure 4). 

**Figure 5:**
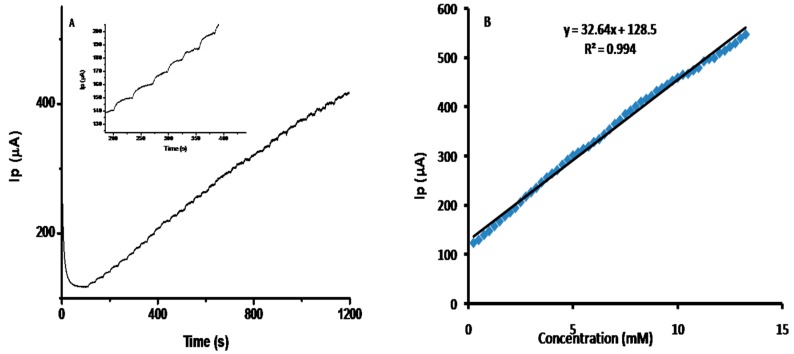
Amperometric current-time response curves for the progressive addition of 0.1 mL aliquots of 100 mM glucose into stirred 0.1 M NaOH at (**A**) a GCE modified with CuO nanostructures; (**B**) Calibration plot currents versus glucose concentrations ranging from 0.25 mM to 13.25 mM.

Amperometry was used to estimate the sensing potential of the electrode modified with newly prepared CuO nanostructures with successive glucose additions in 0.1 M NaOH at an applied potential of +0.5 V (Figure 5A). The solution was stirred continuously. With the addition of glucose, the modified electrode showed rapid and sensitive responses, achieving 90% of the steady-state current within a short interval of time (5 s). The amperometric responses were repeated in triplicate, and the average response was used for the linear range calibration curve (Figure 5B). Calibration curve currents increased with each successive addition of higher glucose concentrations in the linear range of 1 to 10 mM. Greater glucose concentrations saturated the active sites of the CuO nanostructures and thus saturated the current response. The modified electrode sensitivity was found to be 464,285.7 μA·mM^−1^·cm^−2^, which was estimated from the calibration curve to be in the range of 0.25 mM to 13.25 mM (R^2^ = 0.99) and the lower limit of detection was determined (0.0159 mM) for glucose sensing (S/N = 3). The sensitivity of the glucose sensor was calculated by dividing the slope of the calibration curve by the sensing area of the electrode. The high sensitivity and wide linear range was attributed to the high surface-to-volume ratio and the excellent efficiency with which the synthesized CuO nanostructures promoted electron transfer between glucose and the working electrode. Compared to reported enzyme-free sensors based on CuO nanostructures modified electrode, our glucose sensor showed excellent performance in a wide linear detection range (Table 1) [25,26,27,28].

**Figure 6:**
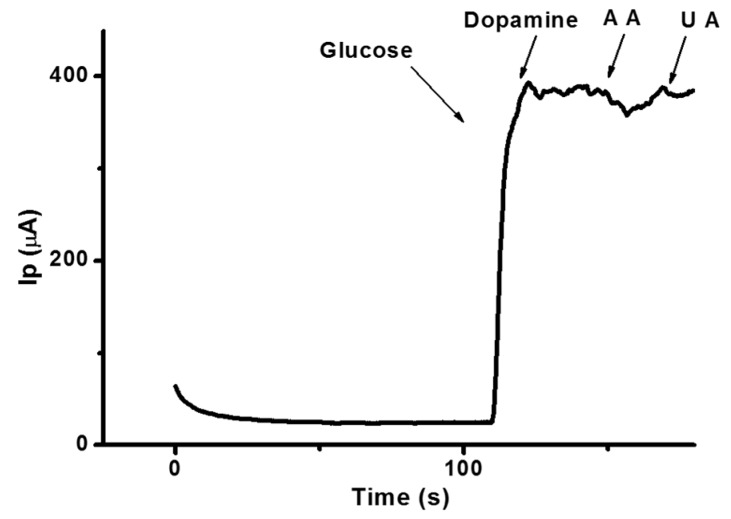
Amperometric current-time response curves for the sequential addition of 0.1 mM aliquots of glucose, dopamine, ascorbic acid, and uric acid.

Selectivity against other endogenous species was assessed to examine any possible glucose sensor signal interference that these species may introduce. The amperometric response of the enzyme-free glucose sensor in 0.1 M NaOH at an applied potential of +0.5 V is shown in Figure 6. The addition of 1 mM glucose to the stirred NaOH solution produced a drastic increase in recorded current. However, the addition of interfering substances such as uric acid, ascorbic acid, and dopamine (0.1 mM each) did not produce a significant amount of current from the fabricated glucose sensor. The lack of interference at the sensor was attributed to the selective property of the synthesized CuO nanostructures. Additionally, the presence of a Nafion membrane layer made it possible for the sensor to exclude interference from these species during glucose sensing [29]. This study confirmed that the fabricated electrode can determine the presence of glucose successfully and with high chemical selectivity, even in samples with dopamine, ascorbic acid, and uric acid interferents. Experiments using different electrochemical modes, such as square wave voltammetry and differential pulse voltammetry, were used to strengthen the results obtained by CV. 

**Figure 7:**
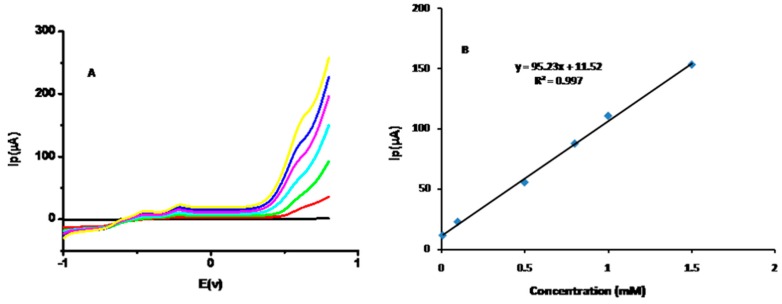
(**A**) Square wave voltammograms for different glucose concentrations: 0.01 mM, 0.1 mM, 0.5 mM, 0.8 mM, 1 mM and 1.5 mM; (**B**) A calibration plot of Ip versus glucose concentration.

Figure 7A shows the square wave voltammograms for different glucose concentrations (0.01 mM, 0.1 mM, 0.5 mM, 0.8 mM, 1 mM, and 1.5 mM). The peak currents increased at the same potential, as discussed with CV measurements. The calibration plot of Ip versus glucose concentration, as obtained from square wave voltammetry, is shown in Figure 7B. The response was clearly linear with different glucose concentrations. 

**Figure 8:**
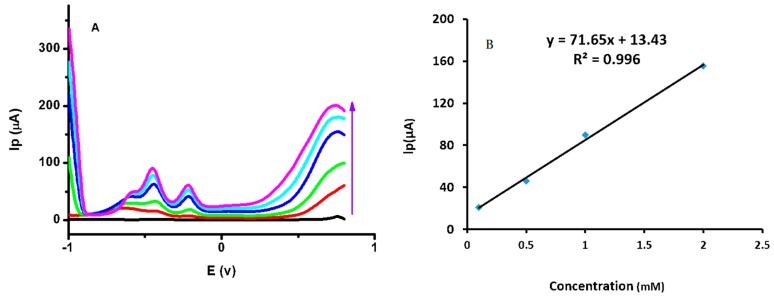
(**A**) Differential pulse voltammograms for different glucose concentrations (0.1 mM, 0.5 mM, 1 mM, 2 mM, and 2.5 mM); (**B**) A calibration plot of Ip versus glucose concentration.

Figure 8A shows the differential pulse voltammograms for different glucose concentrations (0.1 mM, 0.5 mM, 1 mM, 2 mM, and 2.5 mM). A slight shift in glucose oxidation potential was observed, which could be attributed to a less sensitive differential pulse voltammetry response. The calibration plot of Ip versus various glucose concentrations is linear, shown in Figure 8B. These results strongly supported both CV and square wave voltammetry experiments. 

**Figure 9:**
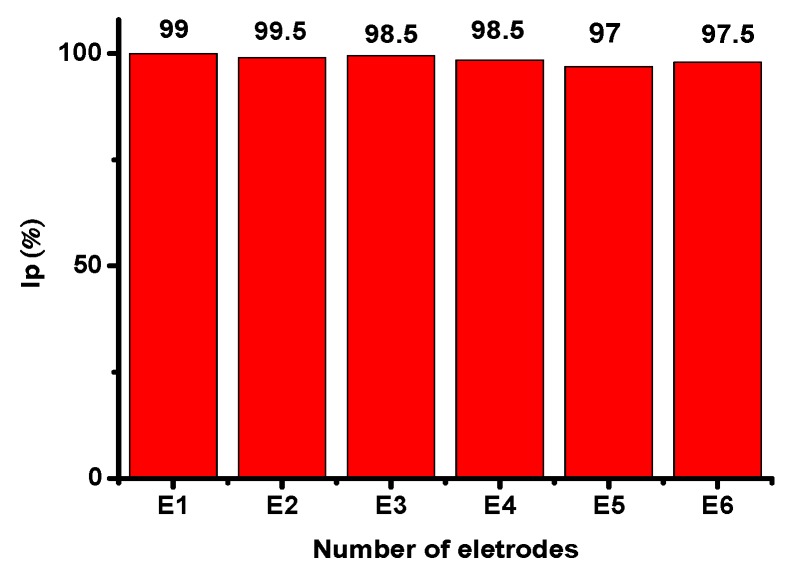
The developed non-enzymatic glucose sensor response was reproducible across electrodes.

The lifetime usability of our developed glucose sensor was monitored by storing the modified electrodes in air under ambient environmental conditions and intermittently measuring the current response during glucose oxidation. The modified electrode was found to retain approximately 98% of the initial current response after a 2-month storage period. Additionally, six modified electrodes were scanned using CV (50 mV/s) in the presence of 0.5 mM glucose to evaluate the glucose sensor reproducibility under similar conditions (Figure 9). The peak currents from CV calibration plot responses from all electrodes were of similar amplitudes, with a low standard deviation (RSD; 3%), indicating that the fabricated glucose sensors were highly reproducible. The potential stability and reproducibility of the modified electrode were indexed to the high chemical stability of the CuO nanostructures synthesized in this study.

To evaluate a practical, routine analysis application for the presented enzyme-free glucose sensor, modified electrodes were utilized to detect the glucose levels in fresh serum samples. Furthermore, The quantified values of glucose in the serum samples obtained using the fabricated glucose sensor using an analytical recovery method were satisfactory, as shown in Table 2. This indicated that the enzyme-free glucose sensor has a practical application in clinical glucose determination. When the modified electrodes were rinsed with deionized water and reused in the serum samples, the sensor replicated approximately 99% of its original response, indicating high sensor reproducibility through repetitive use. The results of the presented CuO-nanostructure-modified GCE were compared with other non-enzymatic glucose sensors reported in the literature, and the performance of our proposed sensor was found to be superior (Table 1). This superiority may be attributed to the unique CuO nanostructure features that were obtained in the presence of lysine as a soft template.

## 4. Conclusions

In this study, a facile approach was adapted in the synthesis of new CuO nanostructures using a hydrothermal method. The synthesized CuO nanomaterial was cost effective and could be used in the development of a robust, enzyme-free glucose sensor. The analytical features of the glucose sensor were highly attractive and included a wide linear range, high sensitivity, quick response time, and low limit of detection. The presented enzyme-free glucose sensor showed high selectivity, stability, and reproducibility. Furthermore, the modified electrodes were used to quantify the glucose concentrations in fresh serum samples. The measured glucose concentrations were found to be similar to those values obtained using analytical quantification. Thus, the presented enzyme-free glucose sensor can be used in a practical application as a tool to determine clinical glucose levels. 

## Figures and Tables

**Table 1:** The comparison of analytical parameters of various reported non-enzymatic glucose sensors with presented glucose sensor.

Types of Electrodes	LOD (μM)	Linear Range (mM)	Sensitivity (μA·μM^−1^·cm^−1^)	Reference
CuO nanorods/G	4.0	4.0–8.0	371.43	[28]
3D Pt nanoporous	N.A	0.1–1.5	642	[30]
Pt Au/C nanocomposite	2	0–10	4.7	[31]
Co_3_O_4_ nanofibers	0.97	Up to 2.4	36.25	[32]
TiO_2_/CNT/Pt/GOx	5.7	0.006–1.5	0.24	[33]
Co_3_O_4_/GCE-Nafion	0.1	0.1–5.0	1618.71	[34]
CuO/GCE-Nafion	0.0159	1–10	464,285.7	This study

**Table 2:** The determination of glucose concentrations in blood serum samples using a CuO-modified GCE.

Sample Number	Conc. of Glucose Added (mM)	Conc. of Glucose Recovered (mM)	Recovery (%)
1	3	3.1	93.1
2	5	4.99	100.2
3	7	7.1	98.59
